# RNA-sequencing and bioinformatics analysis of long noncoding RNAs and mRNAs in the asthenozoospermia

**DOI:** 10.1042/BSR20194041

**Published:** 2020-07-15

**Authors:** Hui Lu, Dongchuan Xu, Ping Wang, Wenye Sun, Xinhuai Xue, Yuxin Hu, Chunli Xie, Yanlin Ma

**Affiliations:** 1 Hainan Provincial Key Laboratory for human reproductive medicine and Genetic Research, The First Affiliated Hospital of Hainan Medical University, Haikou 570102, Hainan, China; 2 Department of Reproductive Medicine, The First Affiliated Hospital of Hainan Medical University, Haikou 570102, Hainan, China; 3 Hainan Provincial Clinical Research Center for Thalassemia, The First Affiliated Hospital of Hainan Medical University, Haikou 570102, Hainan, China; 4 Key Laboratory of Tropical Translational Medicine of Ministry of Education, Hainan Medical University, Haikou 570102, Hainan, China; 5 Haikou Key Laboratory for Preservation of Human Genetic Resource, The First Affiliated Hospital of Hainan Medical University, Haikou 570102, Hainan, China; 6 Department of Emergency, Hainan General Hospital, Haikou 570311, Hainan, China; 7 Division of Biological Sciences, University of California, San Diego, California 92093, U.S.A.

**Keywords:** asthenozoospermia, exosomes, lncRNAs, mRNAs, RNA sequencing

## Abstract

Asthenozoospermia is one of the major causes of human male infertility. Long noncoding RNAs (lncRNAs) play critical roles in the spermatogenesis processes. The present study aims to investigate the intricate regulatory network associated with asthenozoospermia. The lncRNAs expression profile was analyzed in the asthenozoospermia seminal plasma exosomes by RNA-sequencing, and the functions of differentially expressed genes (DEGs) were analyzed by Gene Ontology (GO) and Kyoto Encyclopedia of Genes and Genomes (KEGG) pathway and DO (Disease Ontology) enrichment analyses. Pearson’s correlation test was utilized to calculate the correlation coefficients between lncRNA and mRNAs. Moreover, the lncRNA–miRNA–mRNA co-expression network was constructed with bioinformatics. From the co-expression analyses, we identified the cis regulated correlation pairs lncRNA–mRNA. To confirm sequencing results with five of the identified DElncRNAs were verified with quantitative reverse-transcription polymerase chain reaction (qRT-PCR). We identified 4228 significantly DEGs, 995 known DElncRNAs, 2338 DEmRNAs and 11,706 novel DElncRNAs between asthenozoospermia and normal group. GO and KEGG analyses showed that the DEGs were mainly associated with metabolism, transcription, ribosome and channel activity. We found 254,981 positive correlations lncRNA–mRNA pairs through correlation analysis. The detailed lncRNA–miRNA–mRNA regulatory network included 11 lncRNAs, 35 miRNAs and 59 mRNAs. From the co-expression analyses, we identified 7 cis-regulated correlation pairs lncRNA–mRNA. Additionally, the qRT-PCR analysis confirmed our sequencing results. Our study constructed the lncRNA–mRNA–miRNA regulation networks in asthenozoospermia. Therefore, the study findings provide a set of pivotal lncRNAs for future investigation into the molecular mechanisms of asthenozoospermia.

## Introduction

Infertility is a worldwide health problem, affecting approximately 15% of couples with child-bearing age, and male factor has been deemed to 50% causes for infertility [1]. The main cause of male infertility is sperm quality loss displaying azoospermia, oligozoospermia, asthenozoospermia and teratospermia. In these cases, asthenozoospermia is one of the major causes of human male infertility, characterized by reduced forward motility of spermatozoa (grade A + B sperm motility < 50% or A < 25%), which prevents the sperm from moving to the egg and penetrating it, eventually leading to infertility [2]. However, the cause and pathogenesis of asthenozoospermia are not completely understood.

Exosomes are nanosized extracellular vesicles (40–180 nm) that can be released by almost all types of cells and exist stably in various biological fluids (e.g. blood, urine, saliva, seminal and follicular) [3]. The importance of exosomes lies in their role in intercellular communication by transmitting bioactive molecules, including proteins, DNA, mRNAs and non-coding RNAs. Exosomes could transfer information to recipient cells and thereby influencing the cell functions [4]. These unique properties make exosomes a potential candidate as reliable biomarkers. The recent studies have shown that exosomes are secreted along the male reproductive tract and are thought to be involved in spermatozoa maturation and function [5,6]. Murdica et al. [7] have reported that seminal plasma of men with severe asthenozoospermia contains exosomes that affect spermatozoa motility, and could promptsperm capacitation through an increased induction of tyrosine phosphorylation, therefore, inducing the acrosome reaction. Meanwhile, Murdica et al. [8] revealed the negative modulator of sperm function glycodelin as over-represented in semen exosomes isolated from asthenozoospermic patients by proteomics analysis.

Long noncoding RNAs (lncRNAs) are a group of nonprotein-coding RNAs with a length longer than 200 nucleotides that distributed in the genome broadly [9]. Accumulating evidence suggests that lncRNAs regulate gene expression in the form of RNA in epigenetic regulation, regulation of transcription and post-transcriptional regulation levels [10]. The regulatory and structural functions of lncRNAs have been reported to play pivotal roles in a broad scope of biological processes, and disorderly regulation of lncRNAs leads to diverse human diseases [11]. Recent discovery of lncRNAs as critical regulators in normal and disease development provides new clues for delineating the molecular regulation in male germ-cell development [12]. Necsulea et al. [13] performed a large-scale evolutionary study of lncRNA repertoires and expression patterns, in 11 tetrapod species and found the potential functions for lncRNAs in spermatogenesis processes. Wen et al. [14] revealed that rapidly evolving testis-specific lncRNAs play critical roles in late Drosophila spermatogenesis. Wichman et al. [15] found that dysregulation of specific lncRNAs led to sperm counts or infertility reduced in mice. Recently, Zhang et al. [16] explored the expression profiles and characteristics of human lncRNAs in normal and asthenozoospermia sperm indicating an association between lncRNAs expression and sperm motility. However, the expression and characteristics of lncRNAs in seminal plasma exosomes of normal and asthenozoospermia have not been addressed.

Therefore, to investigate the intricate regulatory network associated with asthenozoospermia, we performed RNA-sequencing and analyzed the differentially expressed genes (DEGs), differentially expressed lncRNAs (DElncRNAs) and differentially expressed messenger RNAs (DEmRNAs) and new lncRNAs between normal and asthenozoospermia groups with bioinformatics in the present study. Subsequently, we predicted the biological function of the DEGs using Gene Ontology (GO) and the Kyoto Encyclopedia of Genes and Genomes (KEGG) pathway and DO (Disease Ontology) enrichment analyses. In addition, we constructed co-expression networks of the lncRNA–miRNA–mRNA with specific bioinformatics approaches to search the hub lncRNAs in the development of asthenozoospermia. To confirm sequencing results with five of the identified DElncRNAs, we performed quantitative reverse-transcription polymerase chain reaction (qRT-PCR) between normal (*n*=20) and asthenozoospermia (*n*=20) groups.

## Materials and methods

### Sample collection

We selected 25 male sterility diagnosed with asthenozoospermia and 25 male normal healthy individuals at the First Affiliated Hospital of Hainan Medical College. The semen samples (8 ml) were collected by masturbation into a sterile container after 3–7 days of abstinence. We used a sperm quality analyzer to assess the semen basic parameters, including morphology, concentration, motility, viability and counts of spermatozoa. According to the World Health Organization (WHO, 2010, Fifth Edition) standards on the diagnostic criteria of asthenozoospermia, the rapid forward progressive motile sperm (grade A <25%) and total progressive motile sperm (grades A + B <50%) in fresh ejaculation [17]. The semen samples were considered normal with the following parameters: sperm concentration ≥ 15 × 10^6^/ml, semen volumes ≥ 1.5 ml, pH ≥ 7.2, progressive motility (PR) ≥ 32%, PR + Non-progressive motility (NP) ≥ 40%. The age range of asthenozoospermia patients and normal healthy controls was 24–48 years old. Control subjects with underlying diseases or abnormal sperm parameters were excluded from the study. All the participants are ethnic Han Chinese with no genetic relationship. Patients with congenital or hereditary disease that cause gonadal dysplasia were excluded from the study. Patients with genitourinary infections, endocrine diseases such as diabetes and hyperthyroidism, severe heart, liver, kidney or other functional abnormalities were also excluded.

### Isolation and identification of exosomes

The semen samples were allowed to liquefy for 30 min at 37°C, 5% CO_2_ incubator. Then, semen samples were centrifuged at 1000 × ***g*** for 10 min to separate seminal plasma containing exosomes. Subsequently, the each seminal plasma sample was transferred to a 15 ml centrifuge tube, and was centrifuged at 300 × ***g*** for 10 min, 2000 × ***g*** for 20 min at 4°C, respectively. We discard the precipitate and remove the cells. Then, supernatants were in turn centrifuged at 10,000 × ***g*** for 30 min. We discard the precipitate and remove subcellular components. The remaining supernatants were centrifuged at 10,000 × ***g*** for 60 min. We discard the supernatant, and the resulting precipitate is the seminal plasma exosomes. Exosome-containing pellets were re-suspended with 30 ml PBS, followed by centrifugation at 10,000 × ***g*** for 60min. The Precipitator-purified exosomes were suspended with 1 ml PBS. Then, samples were loaded separately into the eppendorf tube and stored in a −80°C refrigerator for future use. The morphological characteristics were observed by transmission electron microscopy (H-7650, Hitachi Limited, Japan) according to the manufacturer’s instructions.

### Western blot

Western blot was used to detect exosome-specific antigen molecules CD63 and CD81. We used the reducing Laemmli buffer to dissolve the seminal plasma exosomes (5 μg) and boiled for 5 min at 95°C. Proteins were resolved in a 10% sodium dodecyl sulfate-polyacrylamide gel (SDS-PAGE), followed by being transferred to a PVDF membrane. The membranes were blocked in 5% skimmed milk in PBS containing 0.5% Tween-20 at room temperature for 1 h prior to being probed with the anti-CD63 (1:20,000, #556019; BD Pharmingen), anti-CD81 (1:5,000, #555675; BD Pharmingen) at 4°C overnight. After washing with T-TBS, membranes followed by being incubated with the appropriate horseradish peroxidase-labeled secondary antibody (goat anti-mouse (1: 10,000) or anti-rabbit (1: 2000) immunoglobulin G) for 45 min. The enhanced chemiluminescence (ECL) reagent (Advansta, Menlo Park, CA, U.S.A.) was used to detect the positive immunoreactive bands.

### RNA isolation, library preparation and sequencing analysis

LncRNA-sequencing was performed by the Bioacme Biotechnology Co., Ltd. (Wuhan, China). Briefly, total RNA was extracted with Trizol reagent (Invitrogen, Carlsbad, CA, U.S.A.) from the exosomes of asthenozoospermia patients and normal healthy controls. Total RNA was quantified using a spectrophotometer (NanoDrop 2000; Thermo Fisher Scientific, Waltham, MA, U.S.A.) according to manufacturers’ instructions. We used the Agilent 2100 system and an RNA Nano 6000 Assay kit (Agilent Technologies, Santa Clara, CA, U.S.A.) to assess RNA integrity. The ribosomal RNA (rRNA) was removed using Ribo-Zero rRNA removal beads (Illumina, Inc., San Diego, CA, U.S.A.), fragmentation (the average segment length is approximately 200 nt), to enable accurate lncRNA analysis. The extracted total RNA was reverse transcribed into single-stranded complementary DNA (cDNA), then synthesized into a double-stranded cDNA, terminal repair, 3′ terminal addition, ligation, and addition of primers, PCR amplification and purification, and quality inspection of the library. The generated libraries were sequenced on an Illumina HiSeq 3000 (Illumina Inc, San Diego, CA) with PE150 according to the manufacturer’s protocol. Subsequently, data analyses were performed *in silico*.

### Data processing and quality control

The htseq-count software was used to calculate the gene expression, and the alignment of the mass below 10 in the bam file were excluded, and the obtained gene expression count matrix was used as the downstream analysis data. The raw count data were filtered based on the filter criteria that the counts per million (CPM) was greater than 1. The filtered data were standardized using the R software DESeq2 package. All samples were quality controlled based on standardized data to eliminate outlier samples. We used principal component analysis (PCA) to evaluate inter-sample relationships.

### DEGs analysis

We used the R software DESeq2 package to analyze the differences in gene’s expression levels between the asthenozoospermia group and the normal control group. Genes with *P* value ≤ 0.05 and the log_2_ fold change (FC) absolute value ≥ 1 were considered differentially expressed.

### Enrichment analysis

The GO and KEGG and DO enrichment analyses were applied to determine the functional roles and related pathways of DEGs using the clusterProfiler package in R [18]. The GO analysis (http://www.geneontology.org) is a functional analysis associating DEGs with the GO biological process (BP), cellular component (CC) and molecular function (MF) categories [19]. We make the GO function annotations for DEGs and explore the biological significance of each gene. The KEGG database (http://www.genome.jp/kegg/) includes biochemical reactions, signaling pathways, metabolic pathways and biological processes. KEGG pathway analysis to identify signaling pathways associated with DEGs. The DO (http://disease-ontology.org) analysis was used for the identification of DEG-disease associations [20]. *P* values < 0.05 were considered significantly enriched by the DEGs.

### Association analysis lncRNA and mRNA expression

DElncRNAs and DEmRNA were identified through fold change filtering. The correlation between the expression levels of each DElncRNAs and DEmRNAs was calculated using the Pearson’s correlation test. The correlation coefficient was greater than 0.8 and *P* value less than 0.05 were defined as the DElncRNA–DEmRNA pairs coexpressed. In addition, we performed GO and KEGG enrichment analyses of DElncRNA co-expressing differential genes.

### Target gene prediction

To identify the targeted genes of DElncRNAs, we used the bedtools software to search the nearby coding genes of DElncRNAs and excluded the gene with a distance of more than 100 kilobases upstream or downstream of DElncRNAs. Simultaneously, we took genes that intersect with the co-expressed DEGs with DElncRNAs. These obtained DEGs were defined as the cis-regulated target genes.

### lncRNA–miRNA–mRNA regulatory networks

We used hypergeometric distribution to test whether a DEG target microRNA (miRNA) set was enriched on lncRNA target miRNA set, and *P* value less than 0.05 was considered statistically significant. Accordingly, a co-expression regulatory network representing the possible lncRNA–miRNA–mRNA interaction between key lncRNA and target mRNA/miRNA was established by Cytoscape software (version 3.7.1).

### New lncRNAs prediction

Each sample was assembled separately using Stringtie software, then all sample transcripts were combined using the Stringtie merges command, and then the combined results were compared with known gene models using gffcompare software to discover new transcript information. Moreover, new transcripts with lengths greater than 200 for coding potential identification were screened using the LGC software (http://bigd.big.ac.cn/lgc). Abnormal samples Z1, Z4, R2 were excluded, and we used the R software DESeq2 package for differential analysis to screen differential expression of new lncRNAs.

### qRT-PCR validation of lncRNAs

Trizol reagent (Invitrogen, Carlsbad, CA, U.S.A.) was used to extract RNA from the exosomes of asthenozoospermia patients (*n*=20) and normal healthy controls (*n*=20) according to the manufacturer’s instructions. RNA was reverse transcribed to cDNA using the Reverse Transcription Kit (Takara Co., Ltd., Dalian, China). The quantitative reverse-transcription polymerase chain reaction (qRT-PCR) was performed using the Applied Biosystems 7900HT system (Applied Biosystems) with an SYBR Premix Ex Taq™ kit (Takara Bio, Inc.) according to the manufacturer’s instructions. The primers were designed using Primer BLAST (https://www.ncbi.nlm.nih.gov/tools/primer-blast/) (Supplementary Table S1) and synthesized by Sangon Biotech Co., Ltd. (Shanghai, China). The lncRNA expression level was verified by qRT-PCR using GAPDH as the reference gene with the 2^−ΔΔCT^ method. Each sample was run as three replicates and qPCR experiments were repeated at least three times (*n*=3).

### Statistical analysis

Statistical analysis was carried out using the SPSS13.0 software (IBM Corp, Armonk, NY). The significant differences in expression levels between asthenozoospermia patients and normal healthy controls groups were tested using a two-tailed Student’s *t* test. The significance of the GO terms or enrichment of pathway identifiers in the DEGs was evaluated using the Fisher’s exact test. A value of *P* less than 0.05 was considered statistically significant.

## Results

The basic characteristics and sperm parameters of case and control are summarized in Table 1. A total of 50 subjects were recruited in the case–control study, including 25 patients (mean age ± standard deviation (SD): 32.8 ± 6.53 and 35.0 ± 6.22) and 25 controls (mean age ± SD: 32.4 ± 5.32 and 34.7 ± 5.38). There were no statistically significant differences between case and control group with regarded to age, BMI, smoking and drinking distribution (*P*>0.05). The sperm parameters, including sperm volume, sperm density, PR, IM, and sperm activity rate are significantly different between the case group and the control group (*P*>0.05).

**Table 1:** Basic characteristics and sperm parameters of cases and controls

Variables	Case (*n*=5)	Control (*n*=5)	*P*	Case (*n*=20)	Control (*n*=20)	*P*
Age	32.8 ± 6.53	32.4 ± 5.32	0.918	35 ± 6.22	34.7 ± 5.38	0.871
BMI	24.8 ± 2.69	25.1 ± 3.32	0.884	25.3 ± 3.2	25.6 ± 4.15	0.816
Smoking			0.490			0.736
Yes	3 (60%)	4 (80%)		14 (70%)	13 (65%)	
No	2 (40%)	1 (20%)		6 (30%)	7 (35%)	
Drinking			1.000			1.000
Yes	4 (80%)	4 (80%)		15 (75%)	15 (75%)	
No	1 (20%)	1 (20%)		5 (25%)	5 (25%)	
Sperm volume (ml)	3.0 ± 0.73	4.8 ± 1.28	0.024	2.8 ± 0.8	3.7 ± 1.66	0.041
pH	7.7 ± 0.16	7.7 ± 0.19	0.613	7.5 ± 0.47	7.7 ± 0.17	0.083
Sperm density (×10^6^/ml)	13.9 ± 11.44	59.6 ± 16.58	0.001	13.8 ± 14	55.6 ± 13.57	<0.001
PR	0.1 ± 0.05	0.5 ± 0.04	<0.001	0.1 ± 0.07	0.6 ± 0.04	<0.001
IM	0.9 ± 0.07	0.3 ± 0.13	<0.001	0.9 ± 0.1	0.4 ± 0.02	<0.001
NP	0 ± 0.04	0.1 ± 0.16	0.236	0 ± 0.05	0.1 ± 0.02	0.430
Sperm activity rate	0.1 ± 0.07	0.6 ± 0.02	<0.001	0.1 ± 0.1	0.6 ± 0.03	<0.001

Abbreviations: IM, inactive sperm rate; NP, non-progressive motility; PR, progressive motility.

*P*<0.05 was considered statistically significant.

**Figure 1:**
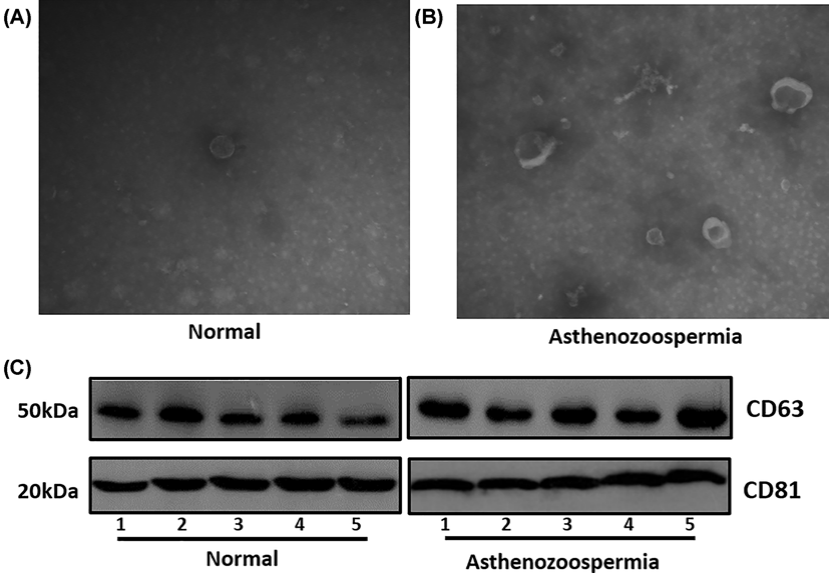
The Characterization of seminal plasma exosomes (**A**) Transmission electron microscope image of exosomes in normal. (**B**) Transmission electron microscope image of asthenozoospermia. (**C**) Western blotting demonstrating the expression of CD63, and CD81 in normal and asthenozoospermia group.

The characterization of exosomes from human seminal plasma was shown in Figure 1. The translucent electron microscope observation results showed that the vesicles derived from normal and asthenozoospermia men displayed similar shape and size, and the ranged of vesicles from 50 to 150 nm, with most of them <100 nm in both groups (Figure 1A,B). To further characterize the vesicles, we used the Western blot to detect the exosome-specific antigen molecules CD63 and CD81 (Figure 1C). The analysis results indicated that all samples expressed these markers, and the total protein content has no significant differences between normal and asthenozoospermia groups.

**Figure 2:**
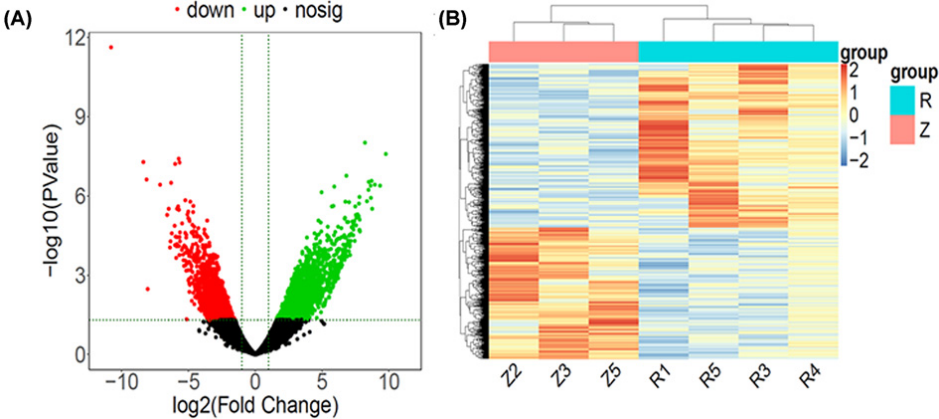
Differentially expressed genes between asthenozoospermia infertility and normal groups (**A**) Volcano plot of DEGs between asthenozoospermia infertility and normal groups. Black dots represent genes that are not significantly differentially expressed. Green and red dots indicate significant up-regulated and down-regulated genes (|log 2 fold change| ≥ 1.0 and *P* value ≤ 0.05), respectively. (**B**) Heatmap of DEGs between asthenozoospermia infertility and normal groups. Row and column were used to represent DEGs and samples. R: asthenozoospermia infertility; Z: normal. The color scale represented the expression levels of DEGs.

We used the R software DESeq2 package to analyze the differences in gene's expression levels between asthenozoospermia and normal groups. A total of 4228 significantly DEGs, containing 2344 up-regulated DEGs and 1884 down-regulated DEGs, in asthenozoospermia group compared with normal group were obtained by |log_2_FC| ≥ 1 and *P*-value ≤ 0.05 (Supplementary Table S2). Top 10 significantly up-regulated and top 10 significantly down-regulated DEGs (asthenozoospermia vs. normal group) were displayed in Table 2. Volcano plots and heatmaps of DEGs between asthenozoospermia and normal groups were displayed in Figure 2A,B.

**Table 2:** The differentially expressed genes between asthenozoospermia and normal (top 10)

No.	Up-regulated			Down-regulated		
	Gene	logFC	*P*-value	Gene	logFC	*P*-value
1	*AC009965.1*	9.767628	2.61E-08	*RN7SL2*	−10.80410	2.43E-12
2	*AL589843.2*	9.334522	4.12E-07	*HMGN1P28*	−8.382749	5.20E-08
3	*AC004853.2*	8.941995	3.76E-07	*AC027309.2*	−8.141726	2.39E-07
4	*AC108751.5*	8.748432	3.08E-06	*AC008021.1*	−8.061548	0.003452
5	*AC010615.1*	8.698275	2.72E-07	*AC011444.3*	−7.130734	3.80E-07
6	*AC121764.1*	8.650715	1.19E-06	*AC009093.4*	−6.598787	5.26E-06
7	*AL138688.2*	8.633034	4.21E-07	*AL359771.1*	−6.485628	3.13E-06
8	*MTCO1P21*	8.493677	2.92E-07	*AL109933.3*	−6.389374	9.01E-05
9	*AL358234.1*	8.473512	5.91E-07	*AL121906.2*	−6.347108	5.36E-05
10	*AC019117.2*	8.350703	1.60E-06	*AC019193.3*	−6.305771	3.27E-07
Total	2344			1884		

Abbreviation: FC, fold change

*P* < 0.05 was considered statistically significant (asthenozoospermia vs. normal group).

**Figure 3:**
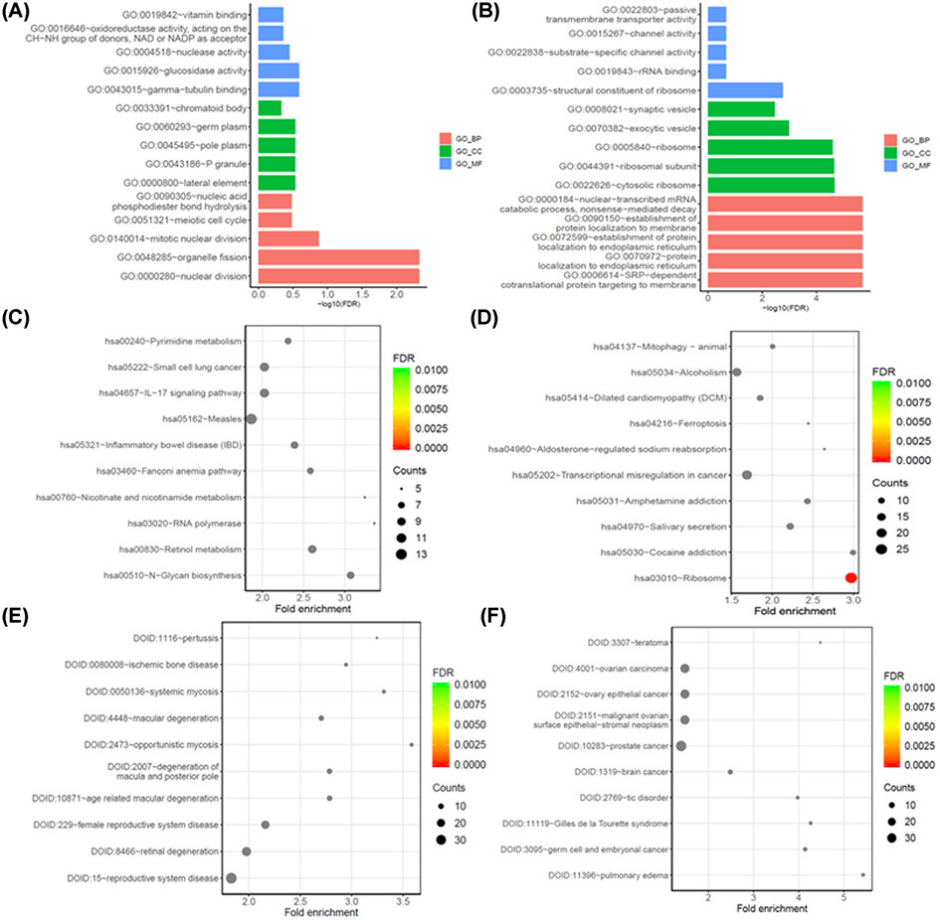
Enrichment analyses of differentially expressed genes (**A** and **B**) Top 5 GO enriched terms for the up-regulated and down-regulated differentially expressed genes, respectively. (**C** and **D**) Top 10 KEGG pathways enriched for the up-regulated and down-regulated differentially expressed genes, respectively. (**E** and **F**) Top 10 DO enriched for the up-regulated and down-regulated differentially expressed genes, respectively; BP, biological process; CC, cellular component; FDR, false-discovery rate; MF, molecular function.

To explore the potential functions of these DEGs, GO and KEGG pathway and DO enrichment analyses were applied with the down-regulated and up-regulated DEGs, separately. Through the GO analysis of DEGs, the up-regulated DEGs were found to be mostly enriched as follows: protein localization to endoplasmic reticulum and establishment of protein localization to membrane ribosome (BP), ribosome and ribosomal subunit (CC), channel activity and passive transmembrane transporter activity (MF) in the top 5 GO enriched terms (Figure 3A and Table 3); the down-regulated DEGs were found to be mostly enriched as follows: nuclear division and organelle fission (BP), lateral element and P granule (CC) and nuclease activity and vitamin binding (MF) in the top 5 GO enriched terms (Figure 3B and Table 3). KEGG pathway analysis revealed that the up-regulated DEGs were mainly associated with ribosome, transcriptional misregulation in cancer, alcoholism, cocaine addiction and ferroptosis in the top 10 KEGG pathways enriched (Figure 3C and Table 4). In addition, the down-regulated DEGs exhibited a strong association with inflammatory and metabolism pathways, according to KEGG pathway analysis (Figure 3D and Table 4). Through the DO analysis, we found that the up-regulated DEGs were significantly enriched among cancers (Figure 3E and Table 5), while the down-regulated DEGs were associated with reproductive system disease, retinal degeneration and mycosis in the top 10 DO enriched (Figure 3F and Table 5).

**Table 3:** GO enrichment analysis of up- and down-regulated DEGs (top 5)

Ontology	Up-regulated DEGs					Down-regulated DEGs				
	ID	Description	Counts	*P*-value	FDR	ID	Description	Counts	*P*-value	FDR
BP	GO:0000280	Nuclear division	48	1.63E-06	0.005	GO:0006614	SRP-dependent cotranslational protein targeting to membrane	25	1.19E-09	1.96E-06
	GO:0048285	Organelle fission	51	1.93E-06	0.005	GO:0070972	Protein localization to endoplasmic reticulum	30	1.20E-09	1.96E-06
	GO:0140014	Mitotic nuclear division	32	8.24E-05	0.135	GO:0072599	Establishment of protein localization to endoplasmic reticulum	27	1.31E-09	1.96E-06
	GO:0051321	Meiotic cell cycle	28	3.52E-04	0.330	GO:0090150	Establishment of protein localization to membrane	48	1.90E-09	1.96E-06
	GO:0090305	Nucleic acid phosphodiester bond hydrolysis	31	4.21E-04	0.330	GO:0000184	Nuclear-transcribed mRNA catabolic process, nonsense-mediated decay	27	1.94E-09	1.96E-06
CC	GO:0000800	Lateral element	5	4.73E-04	0.293	GO:0022626	Cytosolic ribosome	24	3.44E-08	2.18E-05
	GO:0043186	P granule	5	0.002	0.293	GO:0044391	Ribosomal subunit	32	6.96E-08	2.21E-05
	GO:0045495	Pole plasm	5	0.002	0.293	GO:0005840	Ribosome	38	1.19E-07	2.52E-05
	GO:0060293	Germ plasm	5	0.002	0.293	GO:0070382	Exocytic vesicle	29	6.55E-06	0.001
	GO:0033391	Chromatoid body	4	0.005	0.469	GO:0008021	Synaptic vesicle	26	2.73E-05	0.003
MF	GO:0043015	Gamma-tubulin binding	7	0.001	0.259	GO:0003735	Structural constituent of ribosome	29	1.88E-06	0.002
	GO:0015926	Glucosidase activity	5	0.001	0.259	GO:0019843	rRNA binding	12	0.001	0.215
	GO:0004518	Nuclease activity	23	0.001	0.355	GO:0022838	Substrate-specific channel activity	45	0.001	0.215
	GO:0016646	Oxidoreductase activity, acting on the CH-NH group of donors, NAD or NADP as acceptor	5	0.002	0.438	GO:0015267	Channel activity	46	0.002	0.215
	GO:0019842	Vitamin binding	17	0.003	0.438	GO:0022803	Passive transmembrane transporter activity	46	0.002	0.215

Abbreviations: BP, biological process; CC, cellular component; DEG, differentially expressed gene; FDR, false-discovery rate; GO, Gene Ontology; MF, molecular function.

*P*<0.05 was considered statistically significant.

**Table 4:** KEGG pathway enrichment analysis of up- and down-regulated DEGs (top 10)

Up-regulated DEGs					Down-regulated DEGs				
ID	Description	Counts	*P*-value	FDR	ID	Description	Counts	*P*-value	FDR
hsa00510	N-Glycan biosynthesis	8	0.004	0.832	hsa03010	Ribosome	27	2.44E-07	7.09E-05
hsa00830	Retinol metabolism	9	0.007	0.832	hsa05030	Cocaine addiction	9	0.003	0.378
hsa03020	RNA polymerase	5	0.015	0.832	hsa04970	Salivary secretion	12	0.007	0.528
hsa00760	Nicotinate and nicotinamide metabolism	5	0.017	0.832	hsa05031	Amphetamine addiction	10	0.007	0.528
hsa03460	Fanconi anemia pathway	7	0.017	0.832	hsa05202	Transcriptional misregulation in cancer	19	0.017	0.968
hsa05321	Inflammatory bowel disease	8	0.018	0.832	hsa04960	Aldosterone-regulated sodium reabsorption	6	0.024	0.999
hsa05162	Measles	13	0.022	0.832	hsa04216	Ferroptosis	6	0.034	0.999
hsa04657	IL-17 signaling pathway	10	0.025	0.832	hsa05414	Dilated cardiomyopathy	10	0.042	0.999
hsa05222	Small cell lung cancer	10	0.025	0.832	hsa05034	Alcoholism	17	0.044	0.999
hsa00240	Pyrimidine metabolism	7	0.030	0.900	hsa04137	Mitophagy - animal	8	0.044	0.999

Abbreviations: DEG, differentially expressed genes; FDR, false-discovery rate; KEGG, Kyoto Encyclopedia of Genes and Genomes.

*P*<0.05 was considered statistically significant.

**Table 5:** DO enrichment analysis of up- and down-regulated DEGs (top 10)

Up-regulated DEGs					Down-regulated DEGs				
ID	Description	Counts	*P*-value	FDR	ID	Description	Counts	*P*-value	FDR
DOID:15	Reproductive system disease	36	0.000	0.148	DOID:11396	Pulmonary edema	4	0.005	1.000
DOID:8466	Retinal degeneration	26	0.001	0.148	DOID:3095	Germ cell and embryonal cancer	5	0.005	1.000
DOID:229	Female reproductive system disease	21	0.001	0.148	DOID:11119	Gilles de la Tourette syndrome	4	0.012	1.000
DOID:10871	Age-related macular degeneration	10	0.003	0.315	DOID:2769	Tic disorder	4	0.015	1.000
DOID:2007	Degeneration of macula and posterior pole	10	0.003	0.315	DOID:1319	Brain cancer	7	0.020	1.000
DOID:2473	Opportunistic mycosis	7	0.003	0.315	DOID:10283	Prostate cancer	38	0.022	1.000
DOID:4448	Macular degeneration	10	0.003	0.333	DOID:2151	Malignant ovarian surface epithelial-stromal neoplasm	28	0.023	1.000
DOID:0050136	Systemic mycosis	7	0.004	0.373	DOID:2152	Ovary epithelial cancer	28	0.023	1.000
DOID:0080008	Ischemic bone disease	7	0.009	0.503	DOID:4001	Ovarian carcinoma	28	0.023	1.000
DOID:1116	Pertussis	6	0.009	0.503	DOID:3307	Teratoma	3	0.025	1.000

Abbreviations: DEG, differentially expressed gene; DO, Disease Ontology; FDR, false-discovery rate.

*P*<0.05 was considered statistically significant.

**Figure 4:**
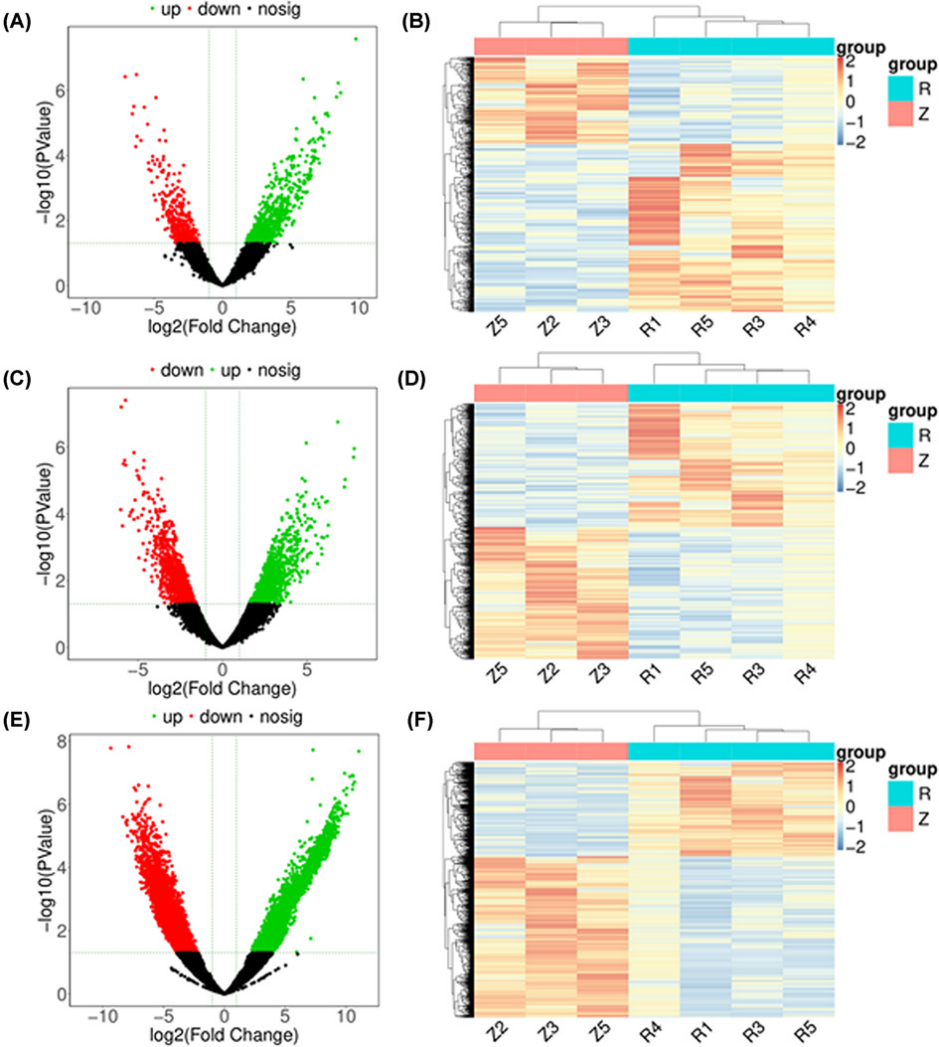
Differentially expressed of lncRNAs and mRNAs between asthenozoospermia infertility and normal groups (**A** and **B**) Volcano plot and heatmap of DElncRNAs between asthenozoospermia infertility and normal groups. (**C** and **D**) Volcano plot and heatmap of DElmRNAs between asthenozoospermia infertility and normal groups. (**E** and **F**) Volcano plot and heatmap of novel DElmRNAs between asthenozoospermia infertility and normal groups. Black dots represent genes that are not significantly differentially expressed. Green and red dots indicate significant up-regulated and down-regulated genes (|log 2 fold change| ≥ 1.0 and *P* value ≤ 0.05), respectively. Row and column were used to represent DElncRNAs/DEmRNAs and samples. R: asthenozoospermia infertility; Z: normal. The color scale represented the expression levels of DElncRNAs/DEmRNAs.

We compared the lncRNA and mRNA expression levels in the asthenozoospermia group with the normal group from the obtained RNA-sequencing data. In total, 995 DElncRNAs were obtained, including 656 up-regulated lncRNAs and 339 down-regulated lncRNAs in the asthenozoospermia compared with the normal control group (Supplementary Table S3). Top 10 significantly up-regulated and top 10 significantly down-regulated DElncRNAs were displayed in Table 5. The volcano plot and heatmaps for clustering analysis of DElncRNAs between asthenozoospermia and normal groups were displayed in Figure 4A,B, respectively. To investigate possible lncRNA–mRNA interactions, we carried out RNA-sequencing analysis and revealed 2338 DEmRNAs between asthenozoospermia and normal control. Among these, 1128 mRNAs were up-regulated in asthenozoospermia group, whereas 1210 mRNAs were down-regulated (Supplementary Table S4). Top 10 significantly up-regulated and top 10 significantly down-regulated DEmRNAs (asthenozoospermia vs. normal) were displayed in Table 5. The volcano plot and heatmaps for clustering analysis of DEmRNAs between asthenozoospermia and normal groups were displayed in Figure 4C,D, respectively. Moreover, we found 11,706 novel DElncRNAs (4321 up-regulated and 7385 down-regulated) in asthenozoospermia group compared with the normal group (Supplementary Table S5, Table 6 and Figure 4E,F).

**Table 6:** The differentially expressed lncRNAs, mRNAs and new lncRNAs (top 10)

	Up-regulated				Down-regulated			
	Symbol	logFC	*P*-value	FDR	Symbol	logFC	*P*-value	FDR
lncRNAs	AC009965.1	9.768	2.61E-08	2.12E-04	AC011444.3	−7.131	3.80E-07	0.001
	PCED1B-AS1	5.908	4.51E-07	0.001	AC019193.3	−6.306	3.27E-07	0.001
	AL358234.1	8.474	5.91E-07	0.001	AC068389.1	−4.871	1.67E-06	0.002
	AC121764.1	8.651	1.19E-06	0.001	AL359771.1	−6.486	3.13E-06	0.002
	AC019117.2	8.351	1.60E-06	0.002	AC008735.2	−5.731	3.30E-06	0.002
	AC112176.1	6.739	1.67E-06	0.002	AC009093.4	−6.599	5.26E-06	0.003
	AC022398.1	7.654	5.01E-06	0.003	LINC02320	−5.486	1.10E-05	0.004
	LSAMP-AS1	7.521	5.33E-06	0.003	AC022098.1	−4.276	1.67E-05	0.006
	AC025300.1	7.738	6.08E-06	0.003	AC022098.2	−6.258	2.60E-05	0.007
	LINC02042	6.713	6.91E-06	0.004	AL513320.1	−4.602	3.38E-05	0.008
Total	656				339			
mRNAs	*PIGC*	6.819	1.74E-07	0.001	*WFIKKN1*	−5.996	6.20E-08	2.16E-04
	*C9orf72*	4.956	7.47E-07	0.001	*HES6*	−5.739	3.88E-08	2.16E-04
	*PPP1R42*	7.797	1.09E-06	0.001	*RPLP2*	−5.240	1.46E-06	0.002
	*KCTD4*	7.750	1.99E-06	0.002	*CRYAB*	−4.645	2.46E-06	0.002
	*SYCP2*	4.721	8.74E-06	0.004	*TMEM178A*	−5.783	2.48E-06	0.002
	*HORMAD1*	7.258	9.28E-06	0.004	*GOLGA6L1*	−5.864	3.15E-06	0.002
	*PRDM5*	4.890	1.03E-05	0.004	*HMGN5*	−5.732	3.41E-06	0.002
	*FRG2B*	7.209	1.55E-05	0.005	*OBP2B*	−4.662	4.11E-06	0.003
	*TATDN1*	4.938	2.21E-05	0.007	*PSCA*	−5.078	5.01E-06	0.003
	*HAPLN1*	4.435	2.66E-05	0.007	*NEFH*	−5.018	7.04E-06	0.004
Total	1128				1210			
Novel lncRNAs	MSTRG.187855	7.291	1.90E-08	2.34E-04	MSTRG.303611	−7.864	1.51E-08	2.34E-04
	MSTRG.33224	11.060	2.11E-08	2.34E-04	MSTRG.84390	−9.343	1.67E-08	2.34E-04
	MSTRG.160782	10.598	1.29E-07	0.001	MSTRG.179148	−7.059	2.49E-07	0.001
	MSTRG.222091	10.538	1.20E-07	0.001	MSTRG.220024	−6.248	2.66E-07	0.001
	MSTRG.282729	10.315	1.33E-07	0.001	MSTRG.200378	−7.405	2.99E-07	0.001
	MSTRG.85233	9.884	1.05E-07	0.001	MSTRG.295090	−7.278	3.64E-07	0.001
	MSTRG.313656	7.236	1.61E-07	0.001	MSTRG.112856	−6.166	6.75E-07	0.001
	MSTRG.171336	10.708	1.96E-07	0.001	MSTRG.1639	−6.257	6.99E-07	0.001
	MSTRG.261274	10.578	2.42E-07	0.001	MSTRG.167804	−6.459	8.44E-07	0.002
	MSTRG.257433	10.073	3.11E-07	0.001	MSTRG.174054	−7.203	8.55E-07	0.002
Total	4321				7385			

Abbreviations: FC, fold change; FDR, false-discovery rate.

*P*<0.05 was considered statistically significant (asthenozoospermia vs. normal group).

To identify the correlation between DElncRNAs and DEmRNAs, we performed the Pearson’s correlation test to calculate the correlation coefficients between lncRNA and mRNAs. The correlation coefficient was greater than 0.8 and *P* value less than 0.05 were defined as the DElncRNA–DEmRNA pairs coexpressed. We found 254,981 positive correlations DElncRNA–DEmRNA pairs. Table 7 showed the top 10 positive correlations DElncRNA–DEmRNA pairs.

**Table 7:** Association analysis of DELncRNAs and DEmRNAs (top 10)

LncRNA	Coding gene	Correlation coefficient	*P*-value
AC080013.5	*APC2*	0.9996	4.47E-09
AC109495.1	*CCDC8*	0.9996	5.63E-09
AL138916.2	*ZFC3H1*	0.9996	6.65E-09
AC006116.9	*IL18R1*	0.9991	5.31E-08
AC005291.2	*ARG1*	0.9988	1.01E-07
AL132639.2	*ANGPTL7*	0.9984	2.00E-07
AL162497.1	*CDC26*	0.9982	2.74E-07
ARHGAP5-AS1	*ZBED6*	0.9980	3.51E-07
AL360270.2	*FAR1*	0.9980	3.53E-07
AL645728.1	*LAD1*	0.9980	3.57E-07

Abbreviation: DE, differentially expressed

Correlation coefficient >0.8 and *P*<0.05 was considered statistically significant.

**Figure 5:**
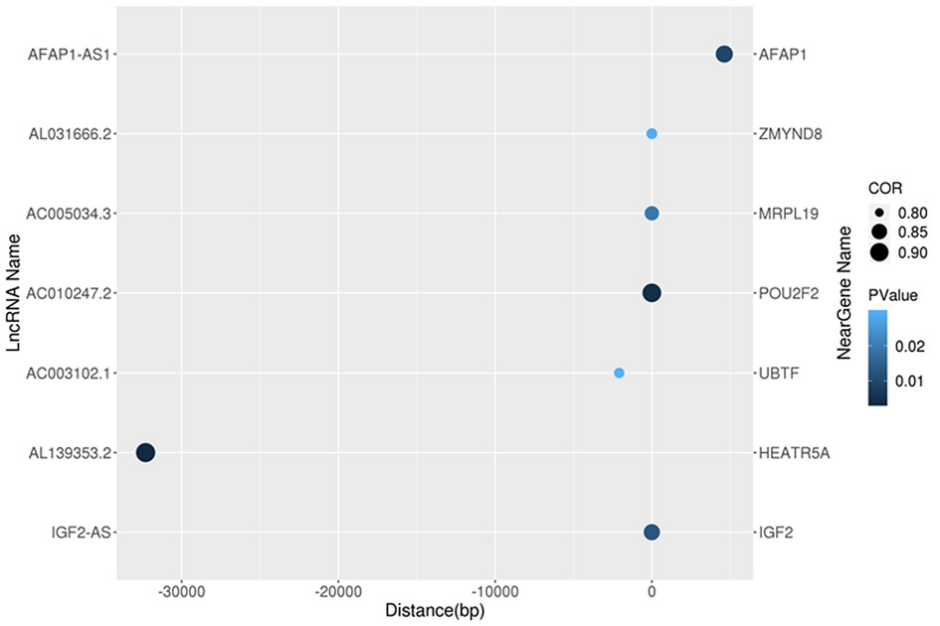
Association of DElncRNAs and cis-regulated target genes The color represents the *P*-value of the correlation analysis. The size of the point represents the magnitude of the correlation coefficient. The horizontal axis is the distance between lncRNA and mRNA.

LncRNA functions by acting on protein-coding genes via cis-acting elements and trans-acting factors. In the present study, to explore how the DElncRNAs may alter sperm function. We predicted the cis- and trans-regulated target genes of DElncRNAs between asthenozoospermia and normal control groups. The closest coding genes to lncRNAs 100 kb upstream and downstream of them were screened, and their associations with lncRNA were analyzed using the Bedtools program. Seven cis-regulated correlation pairs lncRNA–mRNA (IGF2-AS/*IGF2*, AL139353.2/*HEATR5A*, AC003102.1/*UBTF*, AC010247.2/*POU2F2*, AC005034.3/*MRPL19*, AL031666.2/*ZMYND8*, and AFAP1-AS1/*AFAP1*), as showed in the Table 8 and Figure 5.

**Table 8:** The cis-regulated target genes of lncRNAs

LncRNA	Gene	Chromosome	Distance	Correlation coefficient	*P*-value
IGF2-AS	*IGF2*	11	0	0.8613	0.013
AL139353.2	*HEATR5A*	14	−32301	0.9185	0.003
AC003102.1	*UBTF*	17	−2084	0.8036	0.029
AC010247.2	*POU2F2*	19	0	0.9086	0.005
AC005034.3	*MRPL19*	2	0	0.8355	0.019
AL031666.2	*ZMYND8*	20	0	0.8052	0.029
AFAP1-AS1	*AFAP1*	4	4624	0.8771	0.010

Correlation coefficient >0.8 and *P*<0.05 was considered statistically significant.

**Figure 6:**
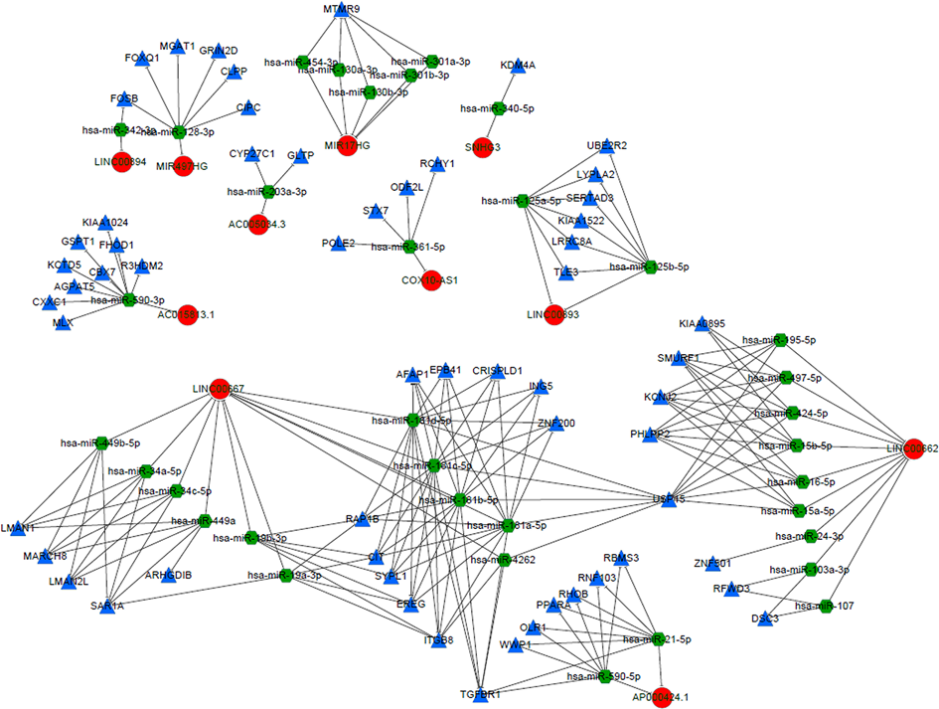
lncRNA–miRNA–mRNA regulatory networks The red circle represents lncRNA, the blue triangle is the co-expressed gene, and the green pentagon represents the miRNA.

We used DElncRNA–DEmRNA co-expression data combined with miRNA target relationship information in the starBase database to perform hypergeometric distribution tests to construct the lncRNA–miRNA–mRNA regulatory network (Figure 6). The interaction network included 11 lncRNAs (LINC00893, AC005034.3, COX10-AS1, MIR497HG, LINC00894, AC015813.1, AP000424.1, MIR17HG, LINC00667, LINC00662 and SNHG3), 35 miRNAs and 59 mRNAs (Supplementary Table S6).

**Figure 7:**
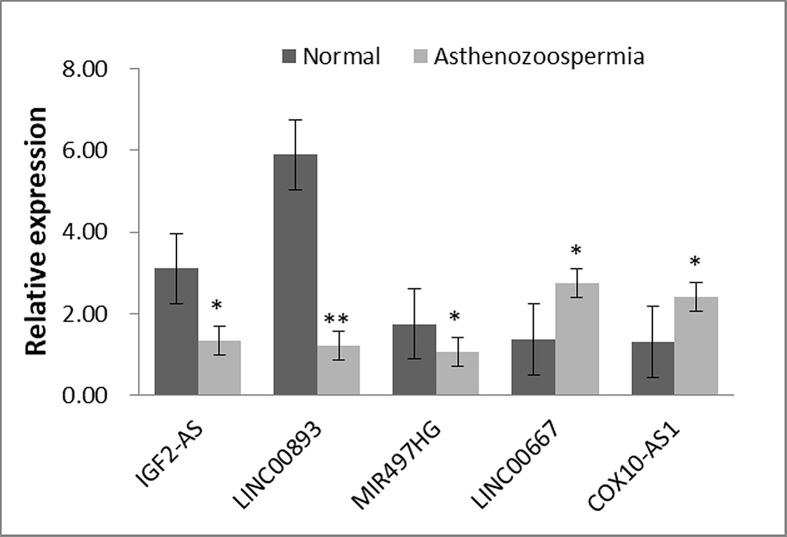
qRT-PCR analysis of five lncRNAs expression between asthenozoospermia infertility and normal groups (*P<0.05; ** *P*<0.001)

To confirm the sequencing and bioinformatics results, two up-regulated (LINC00667 and COX10-AS1) and three down-regulated (LINC00893, MIR497HG and IGF2-AS) lncRNAs in asthenozoospermia group were chosen for qRT-PCR. Consistent with sequencing results, all five lncRNA were found to be differentially expressed in the asthenozoospermia group compared with the normal (*P*<0.05; Figure 7).

## Discussion

In the present study, we performed an RNA-sequencing analysis between asthenozoospermia and normal group and identified 4228 significantly DEGs (2344 up-regulated and 1884 down-regulated) in seminal plasma exosomes RNA. About 995 known DElncRNAs (656 up-regulated and 339 down-regulated) and 11,706 novel DElncRNAs (4321 up-regulated and 7385 down-regulated) and 2338 DEmRNAs (1128 up-regulated and 1210 down-regulated) were identified in the present study. We used the GO and KEGG pathway and DO enrichment analyses to further understand the potential biological functions of these DEGs. Moreover, the lncRNA–miRNA–mRNA co-expression network was constructed, and the result indicated that some hub lncRNAs and mRNAs were involved in asthenozoospermia. From the co-expression analyses, we identified seven cis regulated correlation pairs lncRNA–mRNA. In addition, five of the DElncRNAs were verified with qRT-PCR to further validate the reliability of the RNA-sequencing results.

Exosomes are 40–180 nm double membrane microvesicles originated from cell membrane, which thought to be involved in intercellular communication by transferring information via small biological molecules (i.e. lipids, proteins, DNA, mRNA and noncoding RNA) [21]. On the membrane surface of exosomes display several markers, including *CD9, CD63, CD81, LAMP1* and *TSG101* [21,22]. We detect the antigen molecules *CD63* and *CD81* in the seminal plasma exosomes using the Western blot. The results indicated that *CD63* and *CD81* expressed in all samples, and the total protein content has no significant differences in asthenozoospermia group compared with the normal group. Previous studies indicated that semen exosomes affect spermatozoa motility and functions [7,8].

LncRNAs can regulate gene expression and are abundant in the genomes of various organisms [23]. LncRNAs are usually more than 200 nucleotides in length and exhibit greater species, tissue, and cell specificity than do shorter-length microRNAs (miRNAs) and messenger RNAs (mRNAs) due to their evolutionary unconserved characteristics [24]. The main functions of lncRNAs include roles in chromatin modification, transcriptional regulation and post-transcriptional regulation. In addition, lncRNAs affect the regulation of mRNA translation and stability largely based on the competitive endogenous RNA (ceRNA) regulation mechanism of binding to miRNA. Increasing evidence indicated that the ceRNA regulatory network of lncRNA–miRNA–mRNA was important in different diseases [25,26]. Accumulating evidence indicates that lncRNAs act as regulators in diverse biological processes and play important roles in many human diseases [27]. Moreover, several lncRNAs were reported to serve as disease biomarkers [28].

Recently, Zhang et al. [16] investigated the expression profiles and characteristics of lncRNAs in normal and asthenozoospermia sperm, and indicated the association between lncRNAs expression and sperm motility. In the present study, we performed an RNA-sequencing analysis of asthenozoospermia patients and normal controls seminal plasma exosomes and identified the DEGs, known DElncRNAs, DEmRNAs, the novel DElncRNAs, DEGs functions and involved pathway, and the ceRNA regulatory network of lncRNA–miRNA–mRNA. Seven cis regulated correlation pairs lncRNA–mRNA were identified in the present study.

Previous studies on the insulin-like growth factor II (IGF2) gene are the most widely reported. Spermatozoa is the only source of *IGF2* mRNA since *IGF2* is a paternally inherited gene. Poplinski et al. [29] conclude that low sperm counts were clearly associated with IGF2/H19 ICR1 hypomethylation, and idiopathic male infertility was strongly associated with imprinting defects at IGF2/H19 ICR1. Ni et al. [30] indicated that the aberrant methylation of *IGF2* and *KCNQ1* gene may be associated with sperm DNA damage. Tang et al. [31] revealed that miR-210 was involved in spermatogenesis by targeting *IGF2* in male infertility. Cannarella et al. [32] found that *IGF2* mRNA was found to be present in human spermatozoa, and their transcription levels were positively correlated with sperm concentration and total sperm count.

It has been reported that OCT2 (octamer binding protein 2, POU2F2) acts as a spermatogonial marker to distinguish the early stages of spermatogenesis [33]. *TLE3*, a transcriptional co-regulator that interacts with DNA-binding factors, plays a role in the development of somatic cells. Lee et al. [34] revealed that *TLE3* is differentially expressed in Sertoli cells and plays a crucial role in regulating cell-specific genes involved in the differentiation and formation of Sertoli cells during testicular development. Huang et al. [35] found that *MGAT1* was expressed at lower levels in mouse all germ-cell types, as well as somatic Sertoli cells. Previous studies showed that conditional deletion of the mouse Mgat1 gene (Mgat1 cKO) in spermatogonia causes a germ-cell autonomous defect leading to infertility, and *MGAT1* regulateed ERK1/2 signaling during spermatogenesis via different mechanisms [36,37]. The loss of caseinolytic peptidase P (CLPP) leads to infertility in mice [38]. *EPB41* is one of the pleiotropic monogenic disorder's genes that cause male infertility, and manifests as azoospermia [39]. Bao et al. demonstrated that *LRRC8A*-dependent VRAC activity is essential for male germ cell development and fertility [40]. *LRRC8*/VRAC anion channels are required for late stages of spermatid development in mice [41].

In the present study, our results indicated that the *IGF2, POU2F2, TLE3, MGAT1, CLPP* and *LRRC8A* were co-expressed with lncRNAs IGF2-AS, AC010247.2, LINC00893, MIR497HG, MIR497HG and LINC00893, respectively. These mRNAs expression levels were down-regulated in the asthenozoospermia group compared with the normal group. The *EPB41* and *ODF2L* were co-expressed with lncRNAs LINC00667 and COX10-AS1, respectively. However, the two genes mRNAs expression levels were up-regulated in the asthenozoospermia group. Moreover, the qRT-PCR analysis confirmed our sequencing results. However, further studies are needed to evaluate the roles of DElncRNAs and DEmRNAs in the molecular mechanism of human asthenozoospermia.

There are some limitations that cannot be ignored in the present study. First, the study sample for RNAsequencing was relatively small, which limited the ability to determine the DElncRNA and DEmRNAs between asthenozoospermia and normal group. Further validation analysis with larger sample size and biological test was needed to confirm our conclusions. Second, the biological function study of significantly different lncRNA, mRNA and miRNA was not performed in our research. Therefore, the biological function analyses are required to provide improved understanding of the roles and mechanisms of DElncRNA and DEmRNAs in the pathogenesis of asthenozoospermia.

In conclusion, we identified key DElncRNA and DEmRNAs between asthenozoospermia and normal group. Furthermore, bioinformatics analysis identified the potential functions of DEgenes and the regulatory network was constructed. Our data provide a bioinformatics analysis of genes and pathways that may be involved in the pathological mechanisms of asthenozoospermia. However, further studies are still required to investigate their mechanisms in the occurrence and development of asthenozoospermia.

## Supplementary Material

Supplementary Tables S1-S7

## Data Availability

The datasets used and/or analyzed during the current study are available from the corresponding author on reasonable request.

## Abbreviations

DEG: differentially expressed gene
DO: Disease Ontology
FDR: false-discovery rate
GO: Gene Ontology
KEGG: Kyoto Encyclopedia of Genes and Genomes
lncRNA: long noncoding RNA
MF: molecular function
qRT-PCR: quantitative reverse-transcription polymerase chain reaction
